# Low-Temperature Gas Plasma Combined with Antibiotics for the Reduction of Methicillin-Resistant *Staphylococcus aureus* Biofilm Both In Vitro and In Vivo

**DOI:** 10.3390/life11080828

**Published:** 2021-08-13

**Authors:** Li Guo, Lu Yang, Yu Qi, Gulimire Niyazi, Jianbao Zheng, Ruobing Xu, Xusong Chen, Jingye Zhang, Wang Xi, Dingxin Liu, Xiaohua Wang, Hailan Chen, Michael G. Kong

**Affiliations:** 1 Center for Plasma Biomedicine, State Key Laboratory of Electrical Insulation and Power Equipment, Xi’an Jiaotong University, Xi’an 710049, China; guoli35@mail.xjtu.edu.cn (L.G.); qq460820339@stu.xjtu.edu.cn (Y.Q.); cpb_yan@stu.xjtu.edu.cn (X.C.); zhangjingye2016@stu.xjtu.edu.cn (J.Z.); xiwang1025140102@stu.xjtu.edu.cn (W.X.); xhw@mail.xjtu.edu.cn (X.W.); 2 School of Life Science and Technology, Xi’an Jiaotong University, Xi’an 710049, China; yanglu35@stu.xjtu.edu.cn (L.Y.); gulmira@stu.xjtu.edu.cn (G.N.); xurb@stu.xjtu.edu.cn (R.X.); 3 Department of General Surgery, First Affiliated Hospital of Xi’an Jiaotong University, Xi’an 710061, China; 4 Frank Reidy Center for Bioelectrics, Old Dominion University, Norfolk, VA 23508, USA; h1chen@odu.edu; 5 Department of Electrical and Computer Engineering, Old Dominion University, Norfolk, VA 23529, USA

**Keywords:** low-temperature gas plasma, biofilm, antibiotic, wound, methicillin-resistant *Staphylococcus aureus*

## Abstract

Biofilm infections in wounds seriously delay the healing process, and methicillin-resistant *Staphylococcus aureus* is a major cause of wound infections. In addition to inactivating micro-organisms, low-temperature gas plasma can restore the sensitivity of pathogenic microbes to antibiotics. However, the combined treatment has not been applied to infectious diseases. In this study, low-temperature gas plasma treatment promoted the effects of different antibiotics on the reduction of *S. aureus* biofilms in vitro. Low-temperature gas plasma combined with rifampicin also effectively reduced the *S. aureus* cells in biofilms in the murine wound infection model. The blood and histochemical analysis demonstrated the biosafety of the combined treatment. Our findings demonstrated that low-temperature gas plasma combined with antibiotics is a promising therapeutic strategy for wound infections.

## 1. Introduction

Skin infections represent one of the most common infectious diseases, and microbial infections seriously prevent or delay the healing process [1,2,3]. *Staphylococcus aureus* especially methicillin-resistant *S. aureus* (MRSA), is one of the most frequent causes of skin infections [3,4,5,6,7]. *S. aureus* cells generally form biofilms on infected skin tissues and are not easily eradicated [8,9]. Biofilms formed by aggregated microbial cells are surrounded by a self-produced extracellular polymeric matrix made of proteins, DNA, and polysaccharides, and adhere to a surface, such as the surfaces of living tissues [10,11]. The structure of the biofilm makes the biofilm-associated bacteria more resistant to the host immune defense system, antibiotics, and other antimicrobial agents [12,13,14]. Skin tissues infected with biofilms are generally treated by surgical incision and debridement, topical antimicrobials, administration of antibiotics, or a combination of these treatments [15]. Surgical incision and debridement probably damage the uninfected skin tissues, while the effect of antibiotic treatment is poor and high amounts of antibiotics cause cytotoxicity [16]. Therefore, developing an effective and safe treatment for biofilm reduction is a challenging issue in the therapy of wound infections.

The development of antimicrobials and antibiotic adjuvants is centered around reactive oxygen species (ROS) [17,18,19]. Low-temperature gas plasma (LTGP) contains various reactive oxygen species (ROS) and reactive nitrogen species (RNS), such as H_2_O_2_, ^1^O_2_, ^•^OH, and ^•^NO as well as electrons, ions, and photons. The temperature of LTGP is near room temperature and therefore it treats cells and tissues without thermal damage, reflecting its attractiveness for a range of biomedical applications, such as bacteria inactivation and cancer treatment [20,21,22,23,24]. The FDA has authorized the use of at least three plasma-based products using “plasma biomedicine” technology, and more biomedical applications, such as ulcer treatments, are currently in preclinical and clinical studies [25,26,27]. LTGP treatment can also be used as a novel topical antimicrobial therapy for wound infections. However, high doses of LTGP carry a risk of damaging body tissues, while low doses of LTGP cannot effectively inactivate pathogens, especially when the pathogens are located deep within a tissue [28]. Thus, a combination of topical treatment with a lower dose of LTGP and conventional antibiotics may represent a therapeutic strategy for infectious skin diseases.

In this study, the combination of LTGP and conventional antibiotics were used to treat the methicillin-resistant *S. aureus* biofilms both in vitro and in murine wounds and the combined treatment effectively reduced the *S. aureus* cells in biofilms. These results suggest that this combination is a novel potential treatment strategy for skin infections.

## 2. Materials and Methods

### 2.1. Biofilm Assay

The biofilms were cultured as described previously [9,29]. *S. aureus* ATCC33591, a methicillin-resistant strain, was purchased from the American Type Culture Collection (ATCC). A single *S. aureus* ATCC33591 colony was grown in 4 mL of Tryptic Soy Broth (TSB, Oxoid) at 250 r.p.m. at 37 °C overnight. The overnight *S. aureus* cultures were diluted 1:100 in TSB with 1% glucose. Silica films (10 × 10 × 0.5 mm) were attached to the bottom of wells of 24-well plates, covered with diluted *S. aureus*, and cultured at 37 °C for 3 days. The medium was carefully removed, and the silica films with biofilms were gently washed three times with saline solution (0.9% NaCl). After the different treatments, the biofilms were solubilized in 1 mL of saline in 1.5-mL Eppendorf tubes by sonication for 1 min and vortexing for 5 min. Serial dilutions of each biofilm were performed, and 10 μL of each dilution was spotted onto TSB plates and incubated overnight at 37 °C. The resulting colony-forming units were calculated and analyzed.

### 2.2. LTGP Device and Treatments

**Figure 1:**
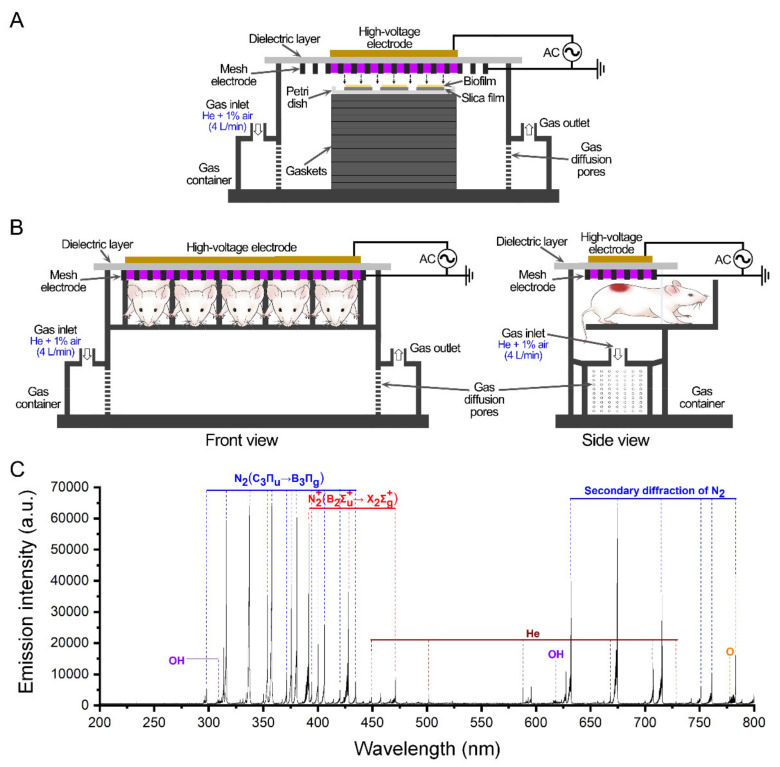
Schematic diagram of the LTGP treatment. (**A**) The treatment of *S. aureus* biofilms with LTGP. (**B**) The front view (left) and side view (right) of the wounds in the murine model treated with LTGP. (**C**) Emission spectra of the surface plasma with the working gas of helium and 1% air.

The LTGP device was similar to that used in previous studies [9,29]. As shown in Figure 1, the surface discharge structure of the LTGP consisted of a high-voltage plane electrode, a liquid-facing grounded mesh electrode with a hexagonal shape, and a dielectric layer (made of polytetrafluoroethylene) sandwiched between the two electrodes. The LTGP was generated when a sinusoidal high voltage was applied, and the discharge power density was 0.2 W/cm^2^ with good mesh-to-mesh homogeneity. The *S. aureus* biofilms in a petri dish (a diameter of 35 mm), which was smaller in size than the LTGP (40 mm × 40 mm), were placed under the LTGP, and the distance between the LTGP and the surface of biofilms was approximately 9 mm (Figure 1A). For the treatment of the wounds in a murine model, the posterior part of the mice was placed under the LTGP, and the distance between the LTGP and the wounds was between 8–12 mm (Figure 1B,C). The LTGP system was housed in a sealed organic glass box with a gas flow of helium and 1% artificial air (79% N_2_ + 21% O_2_) at a constant rate of 4 L/min. Artificial air was used as the source of ROS, while helium was used to enhance the production efficiency of those species as well as their flux on the treated samples via diffusion. The spectra emitted from the plasma were detected by an optical emission spectrometer (Andor, SR750) of which the head of optical fiber was located at 3 cm from the mesh electrode, and the detected spectral range was between 200 and 800 nm.

### 2.3. Antibiotic Treatment of Biofilms

The untreated biofilms and biofilms treated with LTGP were cultured in TSB with 1% glucose and 1250 μg/mL ciprofloxacin, norfloxacin, or vancomycin, or 625 μg/mL rifampicin at 37 °C for 12 h. In the combined experiment of two antibiotics, after culture in TSB containing the first antibiotic at 37 °C for 12 h, the biofilms were washed three times with saline solution and cultured in TSB containing the second antibiotic at 37 °C for another 12 h. The medium was carefully removed, and the biofilms were solubilized in 1 mL of saline in 1.5-mL Eppendorf tubes by sonication for 5 min and vortexing for 1 min. Serial dilutions of each biofilm were performed, and 10 μL of each dilution was spotted onto TSB plates and incubated overnight at 37 °C.

### 2.4. The Minimum Bactericidal Concentration (MBC) Assay

The untreated biofilms and biofilms treated with LTGP were cultured in TSB with 1% glucose and 20–2500 μg/mL ciprofloxacin, norfloxacin, vancomycin, or rifampicin for 24 h. The medium was carefully removed, and the biofilms were solubilized in 1 mL of saline in 1.5-mL Eppendorf tubes by sonication for 5 min and vortexing for 1 min. Serial dilutions of each biofilm were performed, and 10 μL of each dilution was spotted onto TSB plates and incubated overnight at 37 °C.

### 2.5. Regrowth of Biofilms after LTGP Treatment

The biofilms were treated with LTGP for 2, 4, 6, and 8 min, respectively. Then the biofilms were transferred into the fresh TSB containing 1% glucose for regrowth. After culture for 3 days, the biofilms were solubilized in 1 mL of saline by sonication for 5 min and vortexing for 1 min. Serial dilutions of each biofilm were performed, and 10 μL of each dilution was spotted onto TSB plates and incubated overnight at 37 °C.

### 2.6. Mouse Wound Infection Model

Healthy Balb/c mice were purchased from the Laboratory Animal Care Committee of Xi’an Jiaotong University, China. The animals were housed at a temperature of 22 ± 1 °C, relative humidity of 55 ± 5%, and a 12 h dark-light cycle. All experiments in this study complied with the “Guide for the Care and Use of Laboratory Animals.” The dorsal fur of the Balb/c mice was shaved and the skin disinfected by swabbing with 70% ethanol cotton, then the mice were anesthetized with an intraperitoneal injection of sodium pentobarbital. On the depilated posterior part of each mouse, square wounds of 8 × 8 mm on average were generated by cutting carefully through the full thickness of the skin. The animals were randomly divided into six groups, including one uninfected group and five infected groups. Twenty microliters of *S. aureus* ATCC33591 suspension containing approximately 10^8^ cells in saline was inoculated onto each wound, and infections were allowed to progress for 3 days. Uninfected mice were used as additional controls. After 3 days, one group of infected mice was sacrificed and the numbers of bacteria were determined to ensure the establishment of the wound infection model. The infected skin was aseptically excised and homogenized in PBS using a homogenizer (IKA). The homogenates were serially diluted, and samples were plated on TSB agar and incubated at 37 °C overnight.

### 2.7. Treatment

The infected groups were treated individually with LTGP, rifampicin, or a combination of LTGP and rifampicin for 3 days. Photographs of the wounds were taken on days 3, 6, 8, 10, 12, 14, and 16. The wound dimensions were blotted on transparent paper and measured using graph paper. For the treatment of LTGP on the wounds in murine models, the wounds were treated with LTGP for 6 min once every day for 3 days. For the rifampicin treatment groups, rifampicin (30 mg/kg) was given by intragastric administration every 12 h for 3 days. The untreated and treated mice were sacrificed 24 h after treatment. A cardiac blood sample was taken immediately following euthanization and was divided into two tubes: one tube for 0.2 mL was stored in anticoagulant tubes for the hematological test, and another tube was stored at 4 °C overnight, and then the serum was separated by centrifugation for the serum biochemical examination. After the collection of blood, the wound tissue samples were also fixed in 4% (*v/v*) paraformaldehyde for more than 24 h. Then, the fixed tissues were cleared in xylene and embedded in paraffin. To determine the center of the wound and adequately monitor the healing process, the whole sample was serially cross-sectioned (4 µm) with a microtome and mounted on a glass slide. The sections were stained with hematoxylin and eosin. All images were observed under a light microscope and captured at 20× magnification. Anti-*S. aureus* antibody (ab37644, Abcam) was used as the primary antibody in the immunofluorescence analysis and was scanned using a Pannoramic 250 Flash III.

### 2.8. Statistical Analysis

All experiments were performed independently at least three times with triplicate samples. Statistical analyses were performed in SPSS 13.0 (IBM, Armonk, NY, USA) using the Mann–Whitney *U* test of non-parametric statistical tests. The statistical significance of the data was established at a *p*-value of <0.05.

## 3. Results

### 3.1. The Gaseous Reactive Species Produced by the Surface Plasma

The gaseous reactive species produced by the surface discharge plasmas with working gas of helium and 1% air was conducted by optical emission spectrometry (OES). The emission intensities of the N_2_(C_3_Π_u_→B_3_Π_g_) and N_2_^+^(B_2_$\sum_{u}^{+}$→X_2_$\sum_{g}^{+}$) were higher, which resulted from the inelastic collisions between the high-energy electrons in the plasma and the nitrogen, which occupied a large proportion of the ambient air. The trace amounts of oxygen and water vapor in the gaseous phase could also be dissociated to generate excited atoms and molecules such as O and OH and their spectrum lines were diagnosed in the OES. In addition, the spectrum lines of metastable He were also identified. The interaction of these gaseous RONS with aqueous solutions on the surface of biofilms or wounds could induce the production of aqueous reactive species, which directly reacted with the biomolecules and produced the biological effects.

### 3.2. LTGP Treatment Promotes the Reduction of Biofilm by Antibiotics

**Figure 2:**
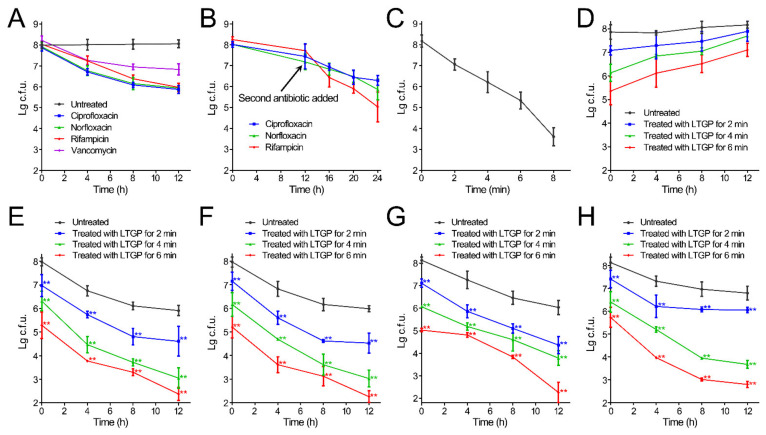
Reduction of *S. aureus* biofilms by LTGP treatment in combination with antibiotics. (**A**) The inactivation of *S. aureus* biofilms by antibiotics. (**B**) The inactivation of *S. aureus* biofilms by two antibiotics sequentially. (**C**) The reduction of *S. aureus* biofilms by LTGP treatment. (**D**) The regrowth of *S. aureus* biofilms after LTGP treatment. (**E**–**H**) The reduction of *S. aureus* biofilms by LTGP treatment in combination with ciprofloxacin (**E**), norfloxacin (**F**), rifampicin (**G**), or vancomycin (**H**). The treated and untreated biofilms were solubilized in 1 mL saline by sonication and vortexing. Serial dilutions of each biofilm were performed and 10 μL of each dilution was spotted onto TSB plates and incubated overnight at 37 °C. Error bars represent the standard deviation (SD). ** *p* < 0.01.

The *S. aureus* biofilms displayed strong tolerance to antibiotics. Treatment with antibiotics for 12 h decreased the number of living cells by less than two orders of magnitude (Figure 2A). Treatment with two antibiotics sequentially decreased the number of living cells by approximately 1–3 orders of magnitude (Figure 2B). LTGP treatment for 6 min decreased the number of living cells by approximately two orders of magnitude and treatment for 8 min decreased the number of living cells by approximately four and a half orders of magnitude, but the cell numbers of the biofilms recovered after 12 h of culture without antibiotics (Figure 2C,D). The treatment of the biofilms using the working gas without applying a voltage exhibited little effect (Supplementary Figure S1). In the experiments of the combination of LTGP and antibiotics, the short-term treatment of LTGP for 2, 4, and 6 min and 1250 μg/mL ciprofloxacin, norfloxacin, or vancomycin, or 625 μg/mL rifampicin, were employed. The combination of LTGP treatment for 6 min and ciprofloxacin, norfloxacin, and rifampicin reduced the living cells in the biofilms close to the detection limit, while the combination of LTGP treatment for 6 min and vancomycin reduced more than five orders of magnitude living cells in the biofilms (Figure 2E–H). Therefore, the combination of LTGP treatment and antibiotics can effectively reduce the living cells in biofilms in vitro.

To further verify the promotion of antibiotic effects by LTGP treatment, the minimum bactericidal concentration (MBC) values of untreated biofilms and biofilms treated with LTGP were measured. The untreated biofilms could be eradicated by only 2500 μg/mL rifampicin. After LTGP treatment for 2 min, the biofilms could be eradicated by 2500 μg/mL ciprofloxacin, norfloxacin, or vancomycin and 1250 μg/mL rifampicin (Table 1). The MBC values of ciprofloxacin, norfloxacin, vancomycin, and rifampicin against biofilms treated with LTGP for 4 min were 625, 1250, 625, and 312.5 μg/mL, respectively, and those against biofilms treated with LTGP for 6 min were 312.5, 312.5, 312.5, and 156 μg/mL, respectively (Table 1). These results indicated that the LTGP treatment lowered the MBC of the S. aureus biofilms and enhanced the killing of living cells in biofilms by antibiotics.

### 3.3. LTGP Treatment in Combination with Rifampicin Reduces Infections in Wounds

**Figure 3:**
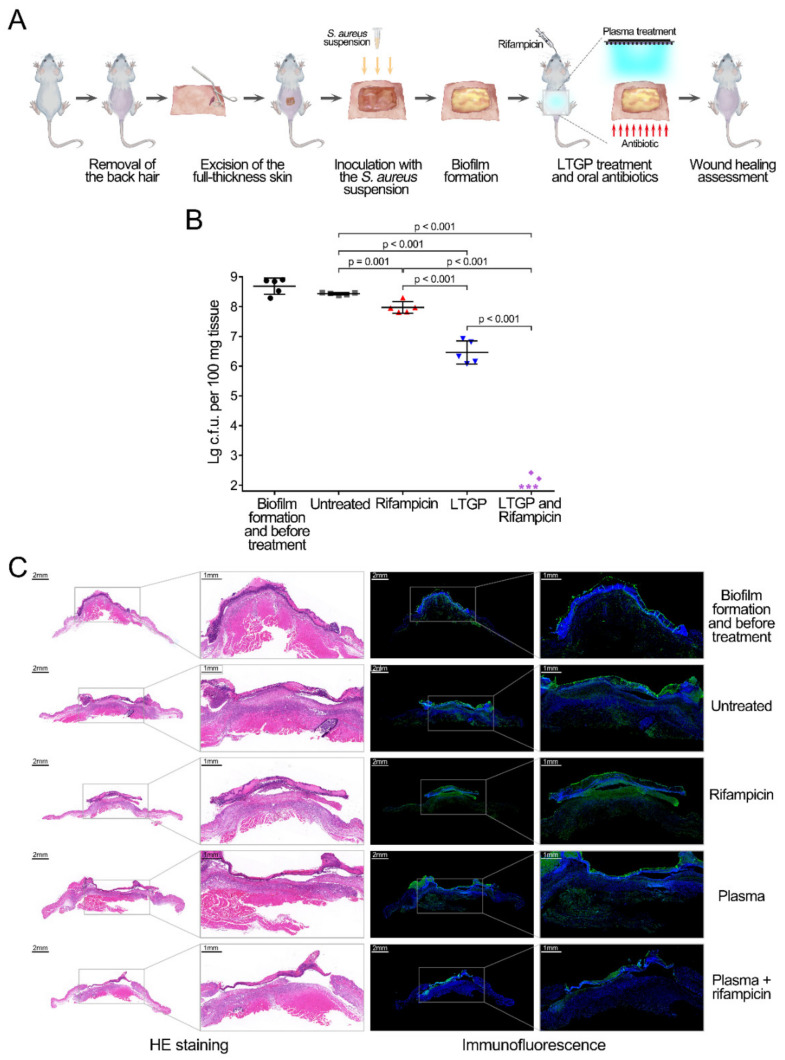
Reduction of *S. aureus* infection in murine wound model by the combined treatment of LTGP and rifampicin. (**A**) Schematic representation of the murine wound model and the treatments with LTGP and rifampicin. (**B**) The reduction of *S. aureus* in infected wounds by the combined treatment of LTGP and rifampicin. Groups of 5 Balb/c mice were used for each experiment. Colony-forming units (c.f.u.) from the wound tissue of each mouse were plotted as individual points, and error bars represent the standard deviation (SD) within an experimental group. The asterisks represent the reduction to the limit of detection. (**C**) Histopathological and immunofluorescence analyses of the skin tissues of the wounds in mice. The right column images of HE staining and immunofluorescence are magnified images of the enclosed area in the left columns. For immunofluorescence analyses, the wound samples were immunostained with anti-*S. aureus* antibody (green) and stained with DAPI (blue).

In an attempt to assess whether LTGP combined with antibiotics could serve as a beneficial tool for wound infection treatment, murine infectious wounds were used to evaluate. The excisional wounds on the posterior parts of mice were infected with methicillin-resistant *S. aureus* and the biofilms formed in the wounds after 3 days. Based on the results of in vitro experiments and administration of antibiotics, rifampicin was selected as the antibiotic for animal experiments. The infected mice were treated with LTGP once a day, intragastric administration with rifampicin every 12 h, or the combination of LTGP and intragastric administration with rifampicin for 3 days (Figure 3A). The reduction of biofilm in wounds was assessed by plate counting and immunofluorescence analysis. The intragastric administration of rifampicin for 72 h reduced less than 0.5-log_10_ *S. aureus* cells and exhibited little effect on the wounds, and the treatment of LTGP alone for 3 days decreased the bacterial cell numbers by approximately 2.0-log_10_ (Figure 3B). Remarkably, the combination of LTGP treatment and rifampicin decreased the bacterial cell numbers by approximately 6.3-log_10_ within 3 days (Figure 3B). Then, the skin tissues around wounds were detected by HE staining and immunofluorescence analysis (Figure 3C). After 3-day treatment, compared with the untreated group, the inflammation reaction in the groups treated with rifampicin alone or LTGP alone and the combined treatment all significantly decreased, especially in the group treated with the combined treatment. The growth of granulation tissue was greatest in the group treated with the combined treatment. In the immunofluorescence analysis, the non-specific binding of the antibody to the scar tissue of the wounds produced non-specific green fluorescence signals on the surface of the wounds (Figure 3C). The *S. aureus* cells represented by green fluorescence signals greatly decreased in the group treated with the combined treatment and decreased in the surface parts in the group treated with LTGP, while that slightly decreased in the group treated with rifampicin, which was consistent with the bacterial counts of wound tissues (Figure 3C). Therefore, the combination of LTGP and rifampicin treatment can effectively reduce biofilms in infected wounds.

**Figure 4:**
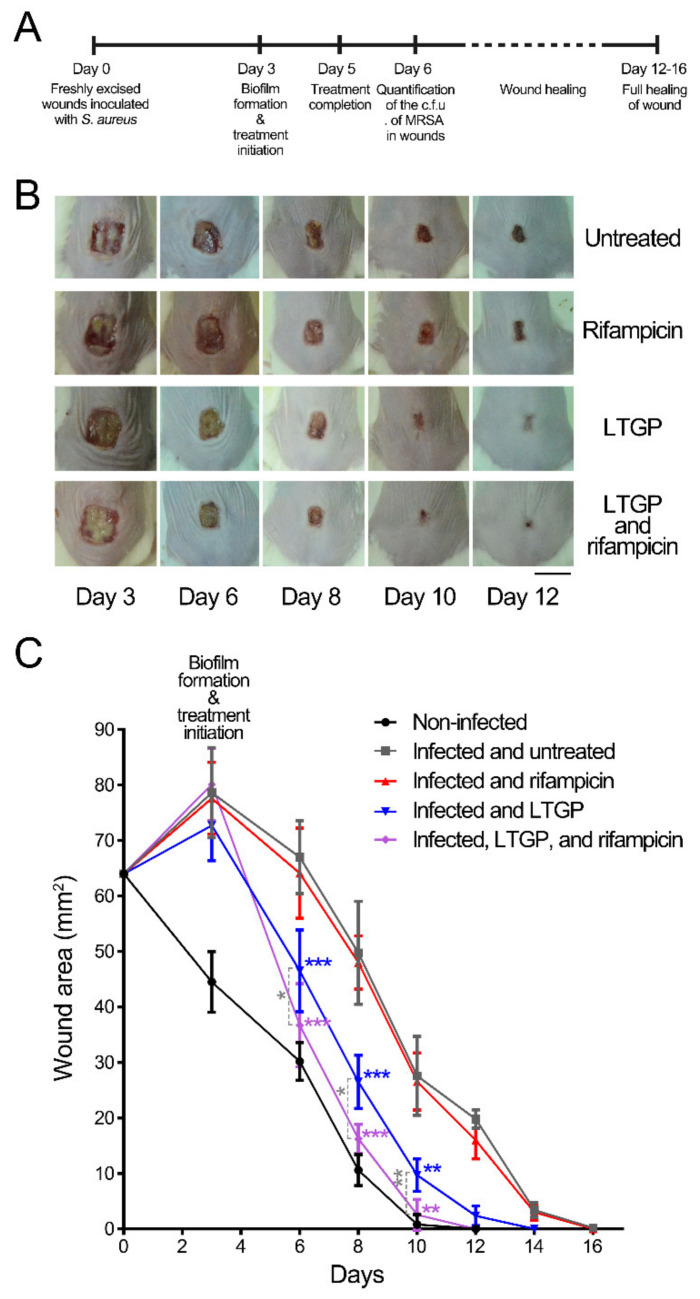
Analysis of wounds in mice. (**A**) A time diagram of wound treatment. (**B**) Wounds of Balb/c mice (*n* = 5 per group) infected with *S. aureus* and then treated with LTGP, rifampicin, or a combination of these two treatments, at day 3, 6, 8, 10, and 12. The bar represents 1 cm. (**C**) Wound sizes of the Balb/c mice skin lesions. Wounds were non-infected, infected and untreated, or infected and treated with LTGP, rifampicin, or a combination of these two treatments. Error bars represent the standard deviation (SD). The results shown are representative of an individual experiment. The blue star represented the *p*-value of the untreated group and the group treated with LTGP, the purple star represented the *p*-value of the untreated group and the group treated with the combination, and the gray star represented the *p*-value of the group treated with LTGP and the group treated with the combination *, *p* < 0.05; **, *p* < 0.01; ***, *p* < 0.001.

The sizes of wounds were measured at day 3, at which the biofilms formed in wounds and the treatment initiated; day 6, at which the bacteria numbers in wounds were determined, and day 8, 10, and 12 (Figure 4A). The wound areas of the non-infected mice decreased and those of the infected mice increased first and then decreased, which was consistent with the phenomenon of a previous study [30]. The sizes of the excisional skin in the wounds treated with LTGP alone or the combination of LTGP and rifampicin reduced more rapidly and could be observed after 6 days, while those of the untreated wounds and the wounds treated with rifampicin groups reduced slowly each day and were not significantly different (Figure 4B,C). The wounds treated with LTGP alone or the combination of LTGP and rifampicin healed significantly quickly and complete wound closure was observed after 10 days, while the untreated wounds and the wounds treated with rifampicin still displayed small amounts of edema and closed after 16 days (Figure 4B,C). Therefore, the combination treatment of LTGP and rifampicin could effectively reduce the biofilms in the infected wounds and promote the healing of the infectious wounds.

### 3.4. Biosafety of the Combined Treatment

**Figure 5:**
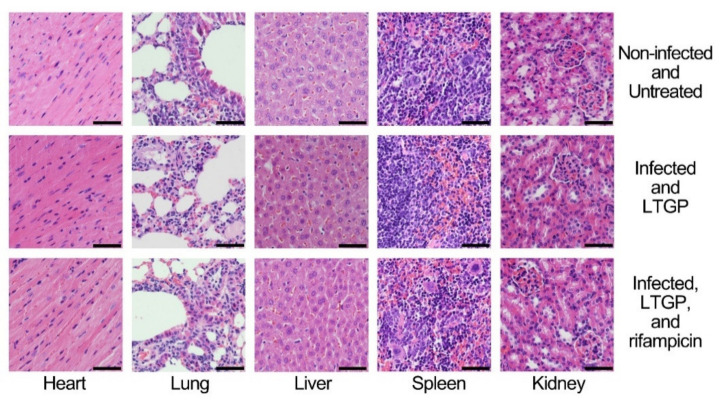
Histopathological analysis of the hearts, lungs, livers, spleens, and kidneys from the infected mice treated with LTGP or the combined treatment of LTGP and rifampicin, and the non-infected and untreated mice. The bars represented 50 μm.

Biological safety should be considered for the application of the combination treatment of LTGP and antibiotics (Table 2). The blood test demonstrated that the group treated with LTGP alone and the group treated with the combination exhibited little change in the numbers of red blood cell count, white blood cell, platelet count, lymphocytes, lymphocytes, monocytes, neutrophils, and the concentration of hemoglobin as compared with the non-infected group. A serum biochemical examination was analyzed and compared for the systemic organ effects. The basic liver function, assessed through the enzyme activity of alkaline phosphate, aspartate aminotransferase (AST), alanine aminotransferase (ALT), and the concentration of total bilirubin in serum showed no significant differences between the group treated with LTGP, the group treated with the combination, and the non-infected group. The kidney function was assessed with the concentrations of blood urea nitrogen (BUN) and creatinine, and the two parameters exhibited no significant differences between the three groups. The levels of albumin, total protein, cholesterol, blood glucose, triglyceride, and uric acid in the serum were also analyzed and there were no significant changes between the three groups (Table 2). Further, a histochemical analysis of the hearts, lungs, livers, spleens, and kidneys from mice was performed. The five organs are commonly used for toxicological tests and the changes in the five organs reflected the toxicity of substances. The comparison showed that the treatment of LTGP alone or the combined treatment with LTGP and rifampicin had little changes of cellular swelling, fatty degeneration, hyaline change, or other pathological damage in the organs (Figure 5). Therefore, the treatment of LTGP alone or the combination treatment of LTGP and rifampicin exhibited little organ toxicity.

## 4. Discussion

Biofilm infections, especially biofilms formed by multidrug-resistant bacteria, are resistant to antibiotics and the immune system; therefore, they are very difficult to cure [31,32]. Because of the toxicities and side effects of conventional antibiotics and the limitations of renal and hepatic function, the effective concentrations of antibiotics for biofilm inactivation are almost impossible to reach [32]. In particular, biofilms formed by antibiotic-resistant bacteria in wounds on the surface of the human body are more difficult to eradicate. Therefore, the combination of effective topical antimicrobials and antibiotics would be a potent strategy for the treatment of wound infections. This study demonstrated that LTGP treatment promoted the effectiveness of antibiotics and lowered the concentrations of antibiotics needed to inactivate MRSA biofilms and that LTGP treatment combined with antibiotics can efficiently reduce biofilms both in vitro and in the murine wound model.

LTGP treatment can lower the MBCs of *S. aureus* biofilms and enhance the reduction of biofilm by antibiotics, and the combination of these two agents effectively treats wound infections caused by antibiotic-resistant bacteria, which may alleviate the increasing antibiotic resistance. A previous study reported that ^•^OH induced in Ag^+^-treated bacteria and O_2_^•‒^ generated by fosfomycin can both potentiate the bactericidal activity of many antibiotics [33,34]. Compared with ROS generated by chemicals, various ROS are generated by the LTGP, and then act directly and synergistically on bacteria without chemical residues. Therefore, LTGP can be developed as a potential topical antimicrobial and combined with antibiotics for the treatment of wound infections.

The intensity of ultraviolet generated in the surface plasma with working gas of Helium and 1% air was was very weak, approximately 3 μW/cm^2^, even lower than the ultraviolet generated in the fluorescent lamp. Therefore, it was supposed that the ultraviolet had little effect and the ROS and RNS played critical roles in the biomedical application [35]. Because of the non-selectivity of reactive oxygen species and reactive nitrogen species, they highly react with biological macromolecules—DNA and proteins—and cause damage. The ROS of LTGP inevitably induced DNA damage, such as double-strand break and DNA-protein crosslinking [9,35,36]. Quinolone antibiotics, ciprofloxacin and norfloxacin, impede DNA replication, which is also blocked by DNA damage [37]. Rifampicin inhibits the transcription process through inactivating RNA polymerase [38]. The ROS of LTGP may also inactivate RNA polymerase and the DNA damage caused by LTGP blocked the transcription process. Vancomycin inhibits bacterial cell wall biosynthesis and the reactive species of LTGP could break the bonds in the cell wall, which could synergize with vancomycin on bacteria [39]. The NO_2_ and ONOO^‒^ of RNS could induce protein damages, such as oligomerization and nitration, and ONOO^‒^ also induced the damage of DNA nucleotide, such as 8-nitroguanine [40,41]. The damages on other enzymes and cell walls caused by ROS and RNS of LTGP can also synergize with different types of antibiotics.

When LTGP is applied to the treatment of wounds, it inevitably acts on the surrounding skin tissues; subsequently, the safety and toxicity of LTGP application should be considered. In this study, the appearance of the skin in healing wounds and the histopathological analysis exhibited no evident changes (Figure 4B and Figure 5). The safety of LTGP has been investigated for a long time, and clinical trials also demonstrated that 5-min daily treatment with LTGP decreased bacteria in chronic wounds of patients without side effects [27]. Studies in animal models showed that LTGP could efficiently inactivate *S. aureus* and *Escherichia coli* on pig skin without inducing morphological changes or damage-related apoptosis [42]. A micronucleus assay on human cells also demonstrated that LTGP treatment did not increase mutagenicity [43]. These studies reflect the safety of LTGP applications in wound treatment.

**Figure 6:**
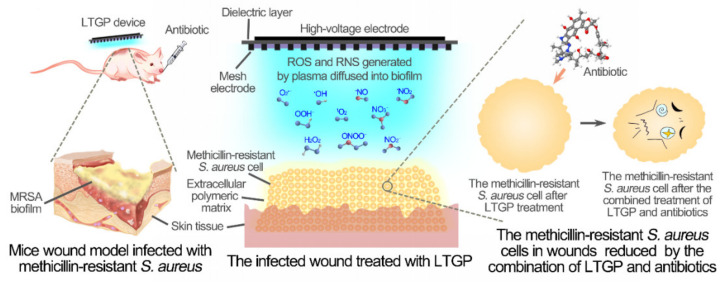
The reduction of methicillin-resistant *S. aureus* in the wound of the murine model by the combined treatment of LTGP and antibiotics.

Based on these results, a model of LTGP treatment combined with antibiotics for the reduction of biofilm was proposed (Figure 6). Reactive oxygen species generated by LTGP, such as O_2_^•−^, diffused into biofilms, entered into *S. aureus* cells and caused damages, which synergize with antibiotics for the reduction of biofilms. The combined application of LTGP treatment and antibiotics could also efficiently reduce biofilms in the murine wound model. Therefore, LTGP treatment combined with antibiotics can effectively reduce biofilms formed by antibiotic-resistant bacteria. Low-temperature gas plasma combined with antibiotics could be developed into a potent therapeutic strategy for wound infections.

## Figures and Tables

**Table 1:** The MBC of the *S. aureus* biofilms after LTGP treatment.

Treatment Time (min)	Ciprofloxacin (μg/mL)	Norfloxacin (μg/mL)	Vancomycin (μg/mL)	Rifampicin (μg/mL)
0	>2500	>2500	>2500	2500
2	2500	2500	2500	1250
4	625	1250	625	312.5
6	312.5	312.5	312.5	156

**Table 2:** Effects on hematological and serum biochemical parameters in mice.

Parameters	Non-Infected and Untreated	Infected and Treated with Plasma	Infected and Treated with Plasma and Rifampicin
Hematology			
Number of red blood cells (×10^12^/L)	11.10 ± 0.56	10.67 ± 0.55	11.45 ± 0.70
Number of white blood cells (×10^9^/L)	4.58 ± 0.68	4.90 ± 1.38 ^a^	5.24 ± 1.92
Platelet count (×10^9^/L)	1263.4 ± 372.2	1254.0 ± 181.0	1224.2 ± 398.2
Hemoglobin (g/L)	167.6 ± 10.6	162.4 ± 8.5	168.2 ± 16.8
Number of lymphocytes (×10^9^/L)	2.18 ± 0.28	2.70 ± 0.67	3.38 ± 1.84
Number of monocytes (×10^9^/L)	0.18 ± 0.08	0.30 ± 0.10	0.24 ± 0.05
Number of neutrophils (×10^9^/L)	1.62 ± 0.64	2.26 ± 0.73	1.62 ± 0.75
Kidney			
Blood Urea Nitrogen (BUN) (mmol/L)	28.70 ± 3.91	26.58 ± 3.13	38.43 ± 14.41
Creatinine (μmol/L)	30.04 ± 1.82	27.33 ± 5.78	31.08 ± 4.11
Hepatic			
Alkaline phosphatase (U/L)	188.13 ± 49.80	161.47 ± 22.69	150.55 ± 47.55
Aspartate aminotransferase (AST, U/L)	209.19 ± 59.78	184.64 ± 40.44	211.25 ± 36.11
Alanine aminotransferase (ALT, U/L)	72.43 ± 38.15	66.06 ± 11.45	74.41 ± 21.99
Total bilirubin (μmol/L)	22.13 ± 4.29	18.08 ± 3.18	20.52 ± 8.23
Nutrition			
Total protein (g/L)	49.36 ± 3.70	45.40 ± 7.49	47.49 ± 3.28
Albumin (g/L)	34.36 ± 3.50	37.26 ± 2.03	33.47 ± 1.05
Cholesterol (mmol/L)	1.82 ± 0.38	2.01 ± 0.19	2.23 ± 0.43
Metabolize			
Triglyceride (mmol/L)	0.57 ± 0.12	0.62 ± 0.10	0.56 ± 0.14
Glucose (mmol/L)	3.05 ± 0.85	3.18 ± 0.71	2.45 ± 0.63
Uric acid (μmol/L)	320.20 ± 92.83	331.79 ± 70.87	349.43 ± 41.62

^a^*p* < 0.05, comparison with the non-infected and untreated group (determined by the Mann–Whitney *U* test).

## Supplementary Materials

The following are available online at https://www.mdpi.com/article/10.3390/life11080828/s1, Figure S1: The effects of the working gas of helium and 1% air (4 L/min) without applying a 2 voltage on the *S. aureus* biofilms.
