# Supplementary material for: Low-Temperature Gas Plasma Combined with Antibiotics for the Reduction of Methicillin-Resistant Staphylococcus aureus Biofilm Both In Vitro and In Vivo
Source: Life (Basel). 2021 Aug 13;11(8):828. doi: 10.3390/life11080828 (PMC8400093; doi:10.3390/life11080828)

Supplementary Information

Supplementary Figures

Supplementary Figure S1. The effects of the working gas of helium and 1% air (4 L/min) without applying a voltage on the *S. aureus* biofilms.

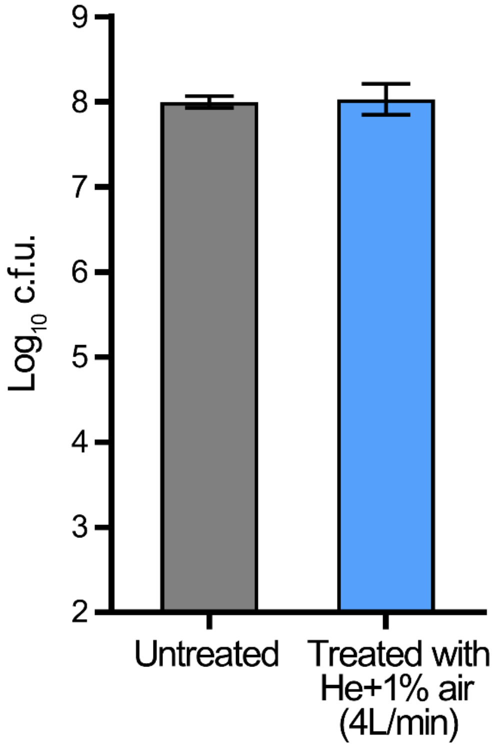

Supplement: Supplementary file 1 [file life-11-00828-s001.zip › life-1320767-supplementary.pdf]
